# Factors Affecting Temporal and Spatial Variations of Microcystins in Gonghu Bay of Lake Taihu, with Potential Risk of Microcystin Contamination to Human Health

**DOI:** 10.1100/tsw.2010.172

**Published:** 2010-09-14

**Authors:** Qing Wang, Yuan Niu, Ping Xie, Jun Chen, Zhimei Ma, Min Tao, Min Qi, Laiyan Wu, Longgen Guo

**Affiliations:** Donghu Experimental Station of Lake Ecosystems, State Key Laboratory of Freshwater Ecology and Biotechnology of China, Institute of Hydrobiology, Chinese Academy of Sciences, Wuhan, China

**Keywords:** microcystins, Gonghu Bay, seasonal variation, Taihu Lake

## Abstract

A field survey of the seasonal variation of microcystin (MC) concentration was performed in Gonghu Bay (a total of 15 sampling sites) of Lake Taihu from January to December 2008. *Microcystis spp.* biomass and intra-/extracellular MCs were significantly correlated with water temperature, suggesting the importance of temperature in cyanobacterial blooming in the lake. Higher MC concentration was found in summer and autumn, and peaks of *Microcystis biomass* and intra-/extracellular MC concentrations were all present in October. Spatially, risk of MCs was higher in littoral zones than in the pelagic area. There were significant correlations between N or P concentrations, and *Microcystis biomass* or MC content, suggesting that N and P levels affected MC production through influencing *Microcystis biomass*. Intra-/extracellular MCs and *Microcystis biomass* had negative exponential relationships with TN:TP, and the maximum values all occurred when TN:TP was <25. Multivariate analyses by pcca indicated that intra- and extracellular MC concentrations had better correlations with biological factors (such as *Microcystis biomass* and chl-a) than physicochemical factors. The maximum concentration reached up to 17 µg/L MC-Lreq, considerably higher drinking water safety standard (1 µg/L) recommended who. So it is necessary take measures reduce exposure risk of cyanobacterial toxins human beings.

## INTRODUCTION

The occurrence of cyanobacterial blooms in eutrophic freshwater bodies has become a worldwide problem[1]. Microcystins (MCs) produced by some cyanobacteria are potent hepatotoxins and tumor promoters by inhibiting protein phosphatase types 1 and 2A[2,3,4,5]. MCs can cause poisoning or death of fish[6], birds[7], and domestic and wild animals[8], as well as illnesses and mortality in humans[9]. They can transfer along the food chain[10], affecting human health through chronic exposure[11].

Cyanobacterial blooms, whether toxic or not, are generally favored by conditions of (1) high light intensities, (2) high temperature levels, (3) high water column stability, and (4) low nitrogen to phosphorus (N:P) ratios[12,13,14,15]. The MC toxicity of a cyanobacterial bloom is determined by both the abundance of toxic cyanobacterial strains and the amount of MCs produced by each cell[16,17,18]. Environmental conditions also indirectly influence MCs through their effects on these two factors. Laboratory and field studies indicated that production of MCs by cyanobacteria is affected by various environmental factors such as light, temperature, and nutrients[19,20,21,22]. Such studies provide valuable information on which environmental conditions most tend to be associated with high MC concentrations; however, empirical relationships between MCs and environmental factors have seldom been developed. Studies on the interactions between MCs and physicochemical variables are still lacking in Chinese lakes in spite of the abundant presence of cyanobacterial blooms in many eutrophic lakes in China[23,24,25].

Lake Taihu is the third largest freshwater lake in China, with regular occurrence of toxic cyanobacterial blooms in warm seasons each year[26]. Its two bays, Gonghu and Meiliang Bays, provide drinking water for millions of residents. In recent years, a few field surveys on MCs have been carried out in Lake Taihu[23,27,28,29,30,31]. However, all studies mentioned above focused on seasonal variations of MCs in Meiliang Bay of Lake Taihu, and only one study has described the seasonal dynamics of MCs at three sampling sites of Gonghu Bay in Lake Taihu[23]. At the end of May 2007, an outbreak of cyanobacterial blooms in Gonghu Bay led to the rapid deterioration of water quality in Gonghu Waterworks, seriously affecting its water supply for 2 million inhabitants of Wuxi city[26]. So it is necessary to monitor the MC pollution in Gonghu Bay. At the same time, understanding environmental factors associated with cyanobacterial bloom formation and MC production is an essential step to predict toxic events and protect public health.

The main purposes of this study were to determine the seasonal dynamics of intra- and extracellular MCs (MC-RR, -LR, -YR) from 15 sites in Gonghu Bay, and to analyze the relationship between MCs and major biological and physicochemical parameters, with discussion on the possible mechanisms underlying these variations and the key factors leading to high MC production. Meanwhile, the potential risk of MCs on human health via drinking water was also evaluated.

## MATERIALS AND METHODS

### Study Area

Lake Taihu (119°54’–120°36’ N, 30°56’–31°33’ E) is located in Jiangsu Province, China. It is characterized by shallowness (mean depth is 1.9 m) and large surface area (2428 $km^2$). It serves as an important resource for drinking water, irrigation, aquaculture, and industrial waters, in addition to being a popular recreational and tourist attraction[29]. Gonghu Bay is an important part of Lake Taihu, which is used as the main drinking water source of Wuxi city. In recent years, this area has been contaminated with a large amount of nutrients and heavy metals, and the water quality of Gonghu Bay has been deteriorating[27]. The occurrence of heavy cyanobacterial blooms in warm seasons has increased in frequency and intensity in recent years, which damages the function of the lake as a drinking water supply, posing a risk to public health[26,32].

### Sample Collection

Fifteen sampling sites (Fig. 1) were set along the northern shore of Gonghu Bay where two waterworks are located. According to the geographical position of the sites, we divided the 15 sites into littoral (Sites

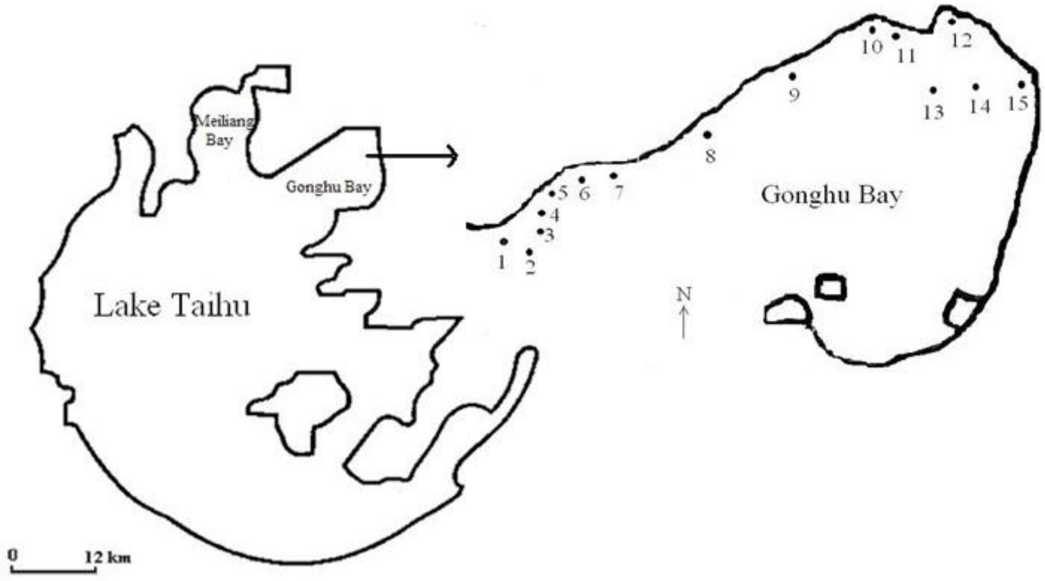

**FIGURE 1.** Sketch map of Gonghu Bay and the sampling sites.

1–12) and pelagic categories (Sites 13–15). Site 3 was near the Nanquan Waterworks, Site 4 was located in a reed marsh, Site 13 was located near the Xidong Waterworks, and Site 15 was near the Wangyu River. The sampling sites were defined by a global positioning system (GPS). Water sampling at each site was an integrated sample collected from the surface and near the bottom every month in Gonghu Bay from January to December 2008.

## The Determination of Water Quality Parameters

Physicochemical and biological water quality–related parameters, including water temperature, pH, water depth, Secchi depth (SD), dissolved oxygen (DO), chemical oxygen demand (COD), total nitrogen (TN), ammonia nitrogen ($NH_4$-N), nitrate nitrogen ($NO_3$-N), nitrite nitrogen ($NO_2$-N), total dissolved nitrogen (TDN), total phosphorus (TP), total dissolved phosphorus (TDP), phosphate phosphorus ($PO_4$-P), chlorophyll a (chl-a), and *Microcystis* biomass were measured for each sample, according to the methods described by Zheng et al.[33].

## Extraction and Determination of Intra- and Extracellular MCs

Lake water (1 L) was filtered with a glass fiber filter (Waterman GF/C, U.K.) to separate toxins dissolved in water (extracellular MCs) and toxins in particles (intracellular MCs). Filter films were extracted three times with 30 mL methanol (75%) and the suspensions were centrifuged at 15,000 rpm (30 min at 4°C). The supernatant was diluted 1:5 with distilled water. The liquor and distilled supernatant were directly concentrated on SPE cartridges ($C_{18}$, 0.5 g), which had been preconditioned by washing with 10 mL methanol (100%) and 10 mL distilled water. Elution from the cartridges with 10 mL methanol (100%) was evaporated to dryness. The residue was dissolved with 100 μL distilled water and used for the qualitative and quantitative analysis of MCs by LC-MS.

Qualitative and quantitative analysis of MCs was performed with a Finnigan LC-MS system comprising a thermo surveyor autosampler, a surveyor MS pump, a surveyor PDA system, and a Finnigan LCQ-Advantage MAX ion trap mass spectrometer equipped with an atmospheric pressure ionization fitted with an electrospray ionization source (LC–ESI-MS). The instrument control, data processing, and analysis were conducted with Xcalibur software (Thermo Electron). Separation was carried out under the reversed phase on an Agilent Zorbax SB-$C_{18}$ column (length, 100 mm; inner diameter, 2.1 mm; film thickness, 3.5 μm). The mobile phase consisted of solvent A [water + 0.05% (v/v) formic acid] and solvent B [acetonitrile + 0.05% (v/v) formic acid]. The linear gradient program was as follows: 0 min at 5% solvent B, 0.5 min at 30% solvent B, 3 min at 40% solvent B, 6 min at 70% solvent B, 14.5 min at 70% solvent B, 14.6 min at 5% solvent B, and 20 min at 5% solvent B. Sample injection volume was typically 10 μL. The MS analytical conditions were as follows: ESI spray voltage 4.54 kV, sheath gas flow rate 20 units, auxiliary gas flow rate 0 units, capillary voltage 3.36 V, capillary temperature 250°C, multiplier voltage –853.19 V, and tube lens offset 55 V. Mass spectrum tuning and optimization were achieved by infusing MC-RR and monitoring the $[M + 2H]^{2+}$ ion at m/z 520. The precursor ion was $[M + 2H]^{2+}$ at m/z 520 for MC-RR, whereas the precursor ion was $[M + H]^{+}$at m/z 995.5 for MC-LR. The limit of detection for the MCs was 0.01 μg/mL.

### Statistical Analysis

To compare variables between the two categories of sites, a student t-test was used. The SPSS (Chicago, IL for Windows (version 13.0) and STATISTIC for Windows statistical software (version 6.0) were used for all analyses.

To characterize the relationships between MCs and environmental variables, we used interval maxima regression (IMR). Based on the descriptions by Graham et al.[34], each variable was divided into equal increments, resulting in 6–16 intervals. The maximum MC value and the associated environmental variable value were obtained from each interval and used in nonlinear regression analysis. IMR relationships were considered significant at $\alpha = 0.05$[34].

Principal component and classifying analysis (PCCA) was used to perform the multivariate relationships among MCs and environmental variables. PCCA transforms a number of possibly correlated variables into a smaller number of uncorrelated principal-components. The first principal-component accounts for the most variability in the data and each accessorial component axis accounts for as much of the remaining as possible[25].

## RESULTS

### *Microcystis* Biomass

*Microcystis* dominated the cyanobacteria community (>99%) throughout the year. Seasonal changes of the *Microcystis* biomass are shown in Fig. 2. *Microcystis* biomass was at a minimum value of 0.0011 mg/L in April, then increased dramatically from the beginning of May and reached peaks (up to 32.1 mg/L) in October. The annual mean value of *Microcystis* biomass was 13.38 mg/L.

### Seasonal Variation of Intra- and Extracellular MC Content

The variations of intra- and extracellular MCs are shown in Fig. 3. Three MC analogues (MC-LR, -RR, and -YR) were identified, among which MC-LR and -RR were the main components. All variants (YR, RR, LR) presented whenever MCs were recorded in the water and phytoplankton in every month of the year, and the proportions (YR:LR:RR) varied from month to month. Intra- and extracellular MC concentrations were both at a low level from January to June, but increased dramatically from July to October when water temperature was above 20°C. Both intra- and extracellular MCs reached peaks (11.678 and 0.167 μg/L, respectively) in October.

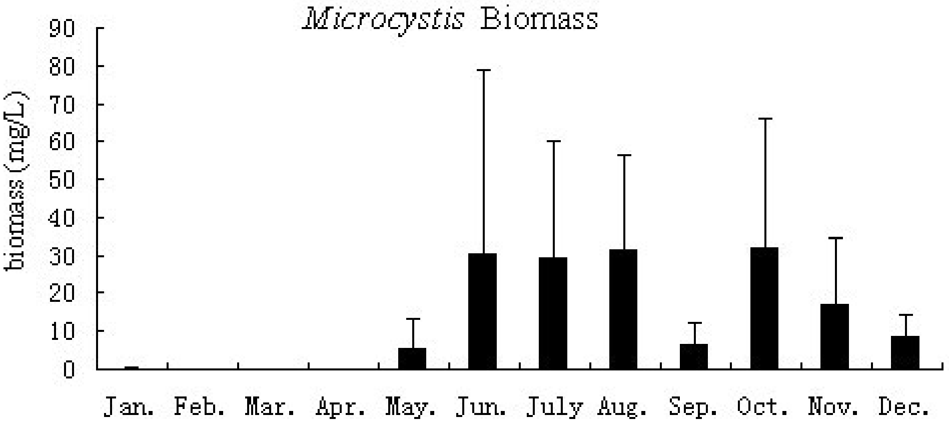

**FIGURE 2.** Seasonal variations of *Microcystis* biomass in Gonghu Bay 2008 (means ± standard deviation).

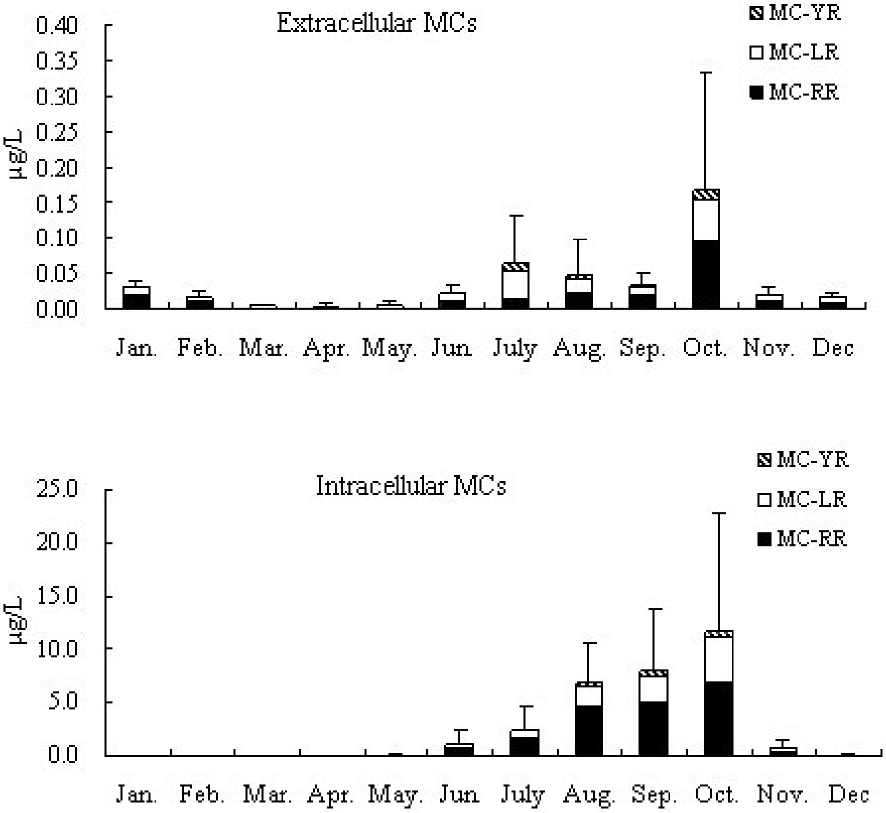

**FIGURE 3.** Seasonal variations of intra- and extracellular MCs (MC-RR+-LR+-YR) in Gonghu Bay 2008 (means ± standard deviation).

## Seasonal Variations of MCs in Representative Sites

Seasonal variations of MCs (MC-RR+-LR+-YR) at Sites 3, 4, 13, and 15 in Gonghu Bay are shown in Fig. 4. We found that the intra- and extracellular MCs of Sites 3 and 4 reached peaks in October, which at Site 13 reached peaks in September. The intracellular MCs of Site 15 reached peak in October and extracellular MCs reached peak in August.

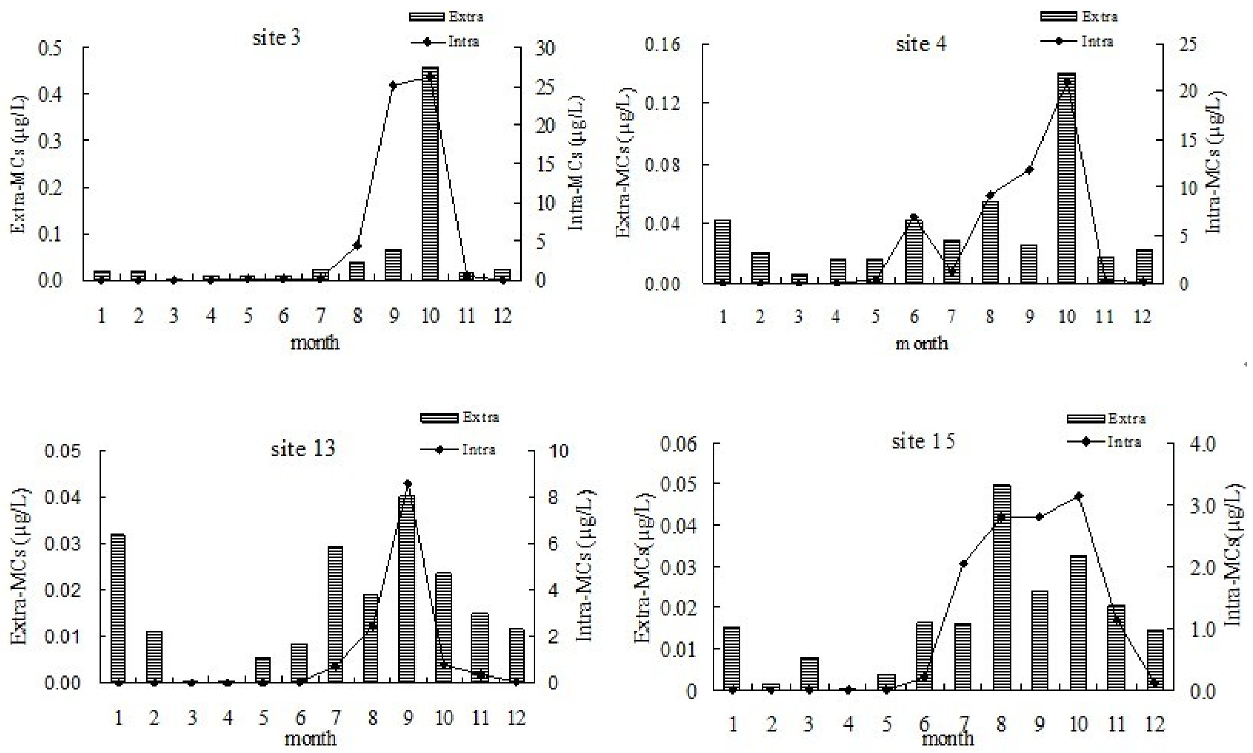

**FIGURE 4.** Seasonal variations of MCs (MC-RR+-LR+-YR) at Sites 3, 4, 13, and 15 in Gonghu Bay 2008.

## Physicochemical Water Quality Parameters

Table 1 shows the mean, minimum, and maximum environmental parameters during the study period. Seasonal dynamics of TN and TP concentrations are illustrated in Fig. 5, from which we could find that TN and TP concentrations both increased from August to October. The TN:TP ratio gradually decreased from January and reached the lowest level (7.27) in September (Fig. 6), then increased continually until December. The maximum value (78 cm) of Secchi depth was observed in December and the minimum value (14 cm) was observed in June (Fig. 7).

## Correlation Analysis

The correlation coefficients between MC concentrations, *Microcystis* biomass, and different environmental factors (water temperature, water depth, SD, DO, pH, chl-a, TN, TDN, $NO_3$-N, $NH_4$-N, $NO_2$-N, TP, TDP, COD, and $PO_4$-P) are listed in Table 2.

The intra- and extracellular MCs were positively correlated with *Microcystis* biomass, TP, DO, TN, water temperature, and chl-a, and negatively correlated with TDN, $NO_3$-N, and SD. In addition, extracellular MCs were positively correlated with COD and negatively correlated with TDP. The *Microcystis* biomass was positively correlated with chl-a, water temperature, TP, and COD, and negatively correlated with $NH_4$, TDN, $NO_3$, and SD.

We obtained the relationship between intra-/extracellular MCs and water temperature by using the mean water temperature of every month as abscissa, and corresponding mean intra- and extracellular MCs as ordinate (Fig. 8). Intra- and extracellular MCs reached peak when water temperature was at 24°C and decreased when water temperature continued to increase.

**TABLE 1**
**Environmental Data for the Gonghu Bay from January to December 2008**

| Parameter | Unit | Mean | Min/Month Occurred | | Max/Month Occurred | |
|---|---|---|---|---|---|---|
| Water temperature | °C | 14.83 | 0.49 | Jan. | 30.81 | Aug. |
| SD | cm | 41 | 14 | June | 78 | Dec. |
| Water depth | m | 1.97 | 1.71 | Jan. | 2.27 | July |
| DO | mg/L | 7.72 | 0.04 | Feb. | 11.03 | Oct. |
| pH | — | 8.35 | 7.88 | Mar. | 9.09 | Oct. |
| COD | mg/L | 5.68 | 3.69 | May | 7.20 | July |
| Chl-a | mg/L | 0.025 | 0.003 | Jan. | 0.06 | June |
| TN | mg/L | 2.28 | 1.21 | Aug. | 3.47 | Mar. |
| TDN | mg/L | 1.68 | 0.42 | Oct. | 3.17 | Mar |
| $NO_3$-N | mg/L | 1.03 | 0.12 | Jan. | 2.57 | Mar |
| $NH_4$-N | mg/L | 0.45 | 0.18 | Oct. | 1.13 | May |
| $NO_2$-N | mg/L | 0.04 | 0.004 | Aug. | 0.20 | Jan. |
| TP | mg/L | 0.15 | 0.06 | Dec. | 0.20 | Oct. |
| TDP | mg/L | 0.04 | 0.02 | July | 0.10 | Mar. |
| $PO_4$-P | mg/L | 0.02 | 0.01 | Dec. | 0.03 | May |

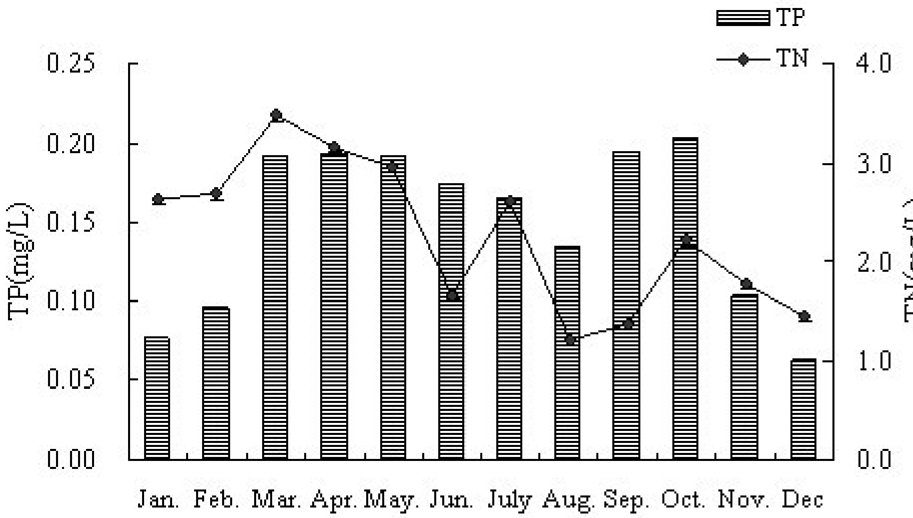

**FIGURE 5**. Seasonal variations of TN and TP concentrations in Gonghu Bay 2008.

## The MC Pollution at Various Sampling Sites

We divided the 15 sites into littoral (Sites 1–12) and pelagic categories (Sites 13–15), and use a student t-test to compare the MCs (intra-MCs + extra-MCs) of every month. We found that MC levels at littoral sites were significantly different ($p$ = 0.009) from the pelagic sites, indicating that the risk of MCs was higher in the littoral zones than the pelagic zones, and the littoral area with relatively less water exchange favored accumulation of cyanobacterial scums and, thus, MCs.

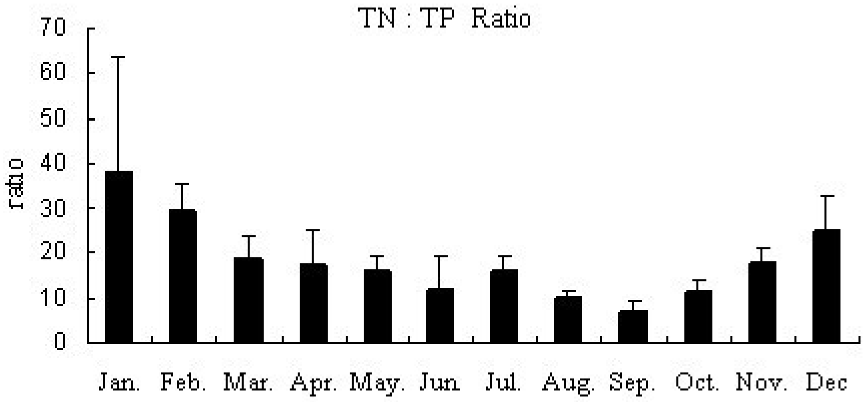

**FIGURE 6.** Seasonal variations of TN:TP ratio in Gonghu Bay 2008 (means ± standard deviation).

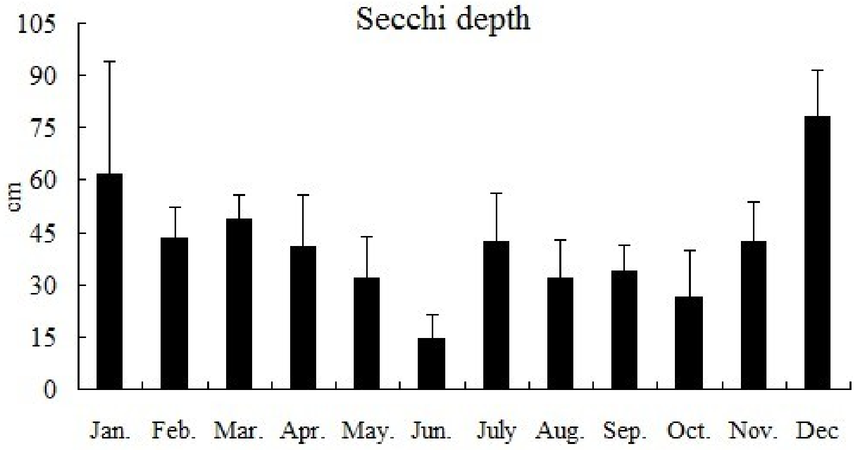

**FIGURE 7.** Seasonal variations of Secchi depth in Gonghu Bay 2008 (means ± standard deviation).

The annual mean concentrations of MCs at each sampling site are shown in Fig. 7. Among the sampling sites, the pollution status of Sites 1–4 was more serious than that of Sites 13–15, and there was nearly a five times difference in the annual mean MC content between the highest and lowest levels (Fig. 9), indicating that risk of MC exposure was much higher to Nanquan Waterworks than to Xidong Waterworks.

## IMR Analysis

Nonlinear IMR indicated that the relationships between the TN:TP ratio and intra- or extracellular MCs could be represented by negative exponential curves (intracellular, r = 0.767, $p < 0.01$; extracellular, r = 0.878, $p < 0.01$, Fig. 8). The relationship between *Microcystis* biomass and the TN:TP ratio could also be described by negative exponential curves (r = 0.785, $p < 0.01$). The maximum intra-/extracellular MCs and *Microcystis* biomass values all occurred when TN:TP ratio was <25 (Fig. 10).

**TABLE 2**
**Correlation Analysis between MC Concentrations, *Microcystis* Biomass, and Environmental Variables**

| | *Microcystis* | Extra-MCs | Intra-MCs |
|---|---|---|---|
| *Microcystis* | 1 | 0.431(**) | 0.457(**) |
| $NH_4$ | –0.157(*) | –0.107 | –0.106 |
| $NO_2$ | 0.104 | –0.094 | –0.09 |
| $NO_3$ | –0.247(**) | –0.228(**) | –0.183(*) |
| TDN | –0.315(**) | –0.348(**) | –0.296(**) |
| TN | 0.032 | 0.152(*) | 0.185(*) |
| $PO_4$ | –0.125 | –0.077 | –0.057 |
| TDP | –0.041 | –0.156(*) | –0.101 |
| TP | 0.215(**) | 0.426(**) | 0.555(**) |
| COD | 0.314(**) | 0.275(**) | 0.081 |
| DO | 0.132 | 0.253(**) | 0.224(**) |
| Chl-a | 0.730(**) | 0.380(**) | 0.455(**) |
| Temp | 0.380(**) | 0.341(**) | 0.343(**) |
| SD | –0.345(**) | –0.246(**) | –0.264(**) |
| WD | –0.005 | –0.017 | –0.023 |

* Correlation is significant at the 0.05 level (two-tailed).

** Correlation is significant at the 0.01 level (two-tailed).

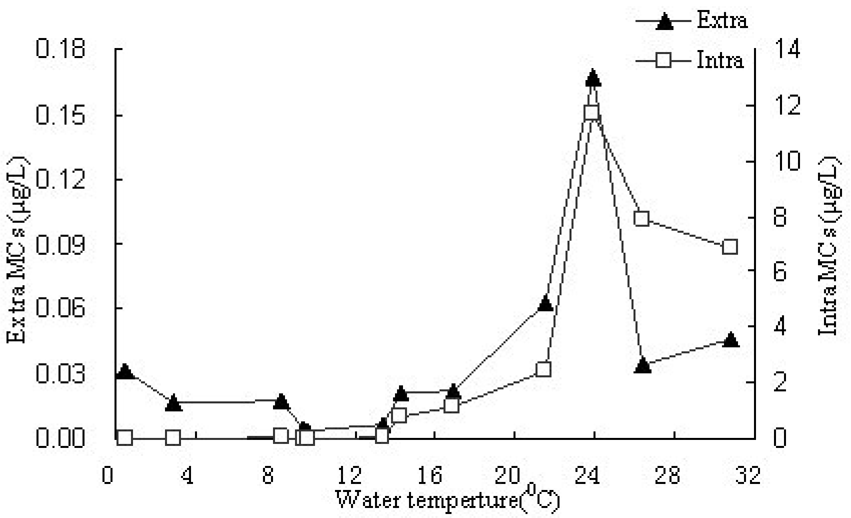

**FIGURE 8.** Relationship between water temperature and intra- or extracellular MCs.

## PCCA

With the PCCA (Fig. 11), more than 50% of the environmental and biological variation in the data was explained by Component Axis 1 (33.38%) and Component Axis 2 (20.36%). Component Axis 1 had high positive weighting for SD and some chemical variables (such as N, P), but negative weighting for water

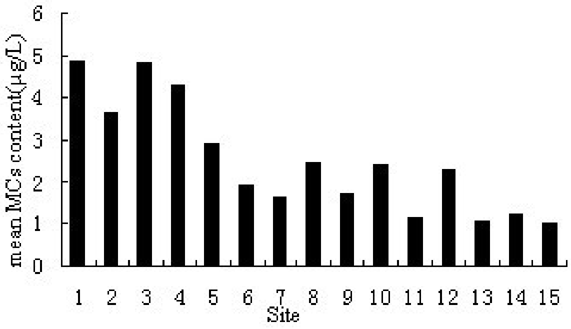

**FIGURE 9.** Annual mean MC concentrations in each sampling site (cf. map in Fig. 1).

temperature and some biological variables (such as *Microcystis* biomass, chl-a). Component Axis 2 had high positive weighting for most biological and chemical variables, but negative weighting for SD. *Microcystis* biomass and MCs (intra- and extracellular) had negative weightings on Component Axis 1 and positive weightings on Component Axis 2.

## DISCUSSION

It may be easy to find empirical relationships between environmental variables (biological and physicochemical) and cyanobacterial biomass[12,13,14,15], but it is not the same for MCs. The overall concentration of MCs in a bloom is determined by both the cellular rates of MC production and community dynamics of cyanobacterial populations. Environmental control may be the result of control of cyanobacterial growth rate, which in turn is correlated with cellular MC content[16]. Natural cyanobacterial populations experience frequent and rapid fluctuations in growth resources from limitation to optimal conditions, and in large and complex water bodies (e.g., Lake Taihu), water movements can affect the distribution of MCs and make it difficult to relate MC concentration with some environmental factors[35], which may explain our findings in Gonghu Bay that some factors (such as $NH_4$) have good correlation with *Microcystis* biomass, but had no correlation with MCs.

*Microcystis* biomass and intra-/extracellular MCs were significantly correlated with water temperature in our study, suggesting that breakout of cyanobacterial blooms and MC production by *Microcystis* was closely related to temperature. It seems that higher temperatures might increase the biomass of toxin-producing *Microcystis* and the production of MCs[27]. In the present study, higher water temperatures in summer and autumn, therefore, explain why there were higher MC concentrations in Gonghu Bay, and the maximum value of intra- and extracellular MCs occurred at 24°C (in October). The optimal temperature for MC production by *M. aeruginosa* was between 20 and 25°C in previous laboratory[36,37] and field studies[24]. Our results are in agreement with these studies.

In our study, intra- and extracellular MCs were positively correlated with TN and TP, which was consistent with a study in the U.S.[34], but inconsistent with studies in Canada[38] and Germany[22]. As MC concentrations in field environments are affected both directly by MC-producing cyanobacteria and indirectly by environmental variables, and these variables vary temporally and spatially, it is not surprising to see the lack of consistent empirical relationships between MCs and environmental variables. At the same time, the cyanobacterial biovolume loss through processes such as sedimentation, grazing, parasitism, or dilutions, processes which vary with season and species, are therefore difficult to quantify[39]. In the present study, there were significant correlations among N or P concentrations, *Microcystis* biomass, and MC content, suggesting that N and P levels affected MC production through influencing *Microcystis* biomass. Kotak et al.[38] also indicate an effect of TP on MC concentration through an indirect effect of TP on the biomass of *Microcystis*.

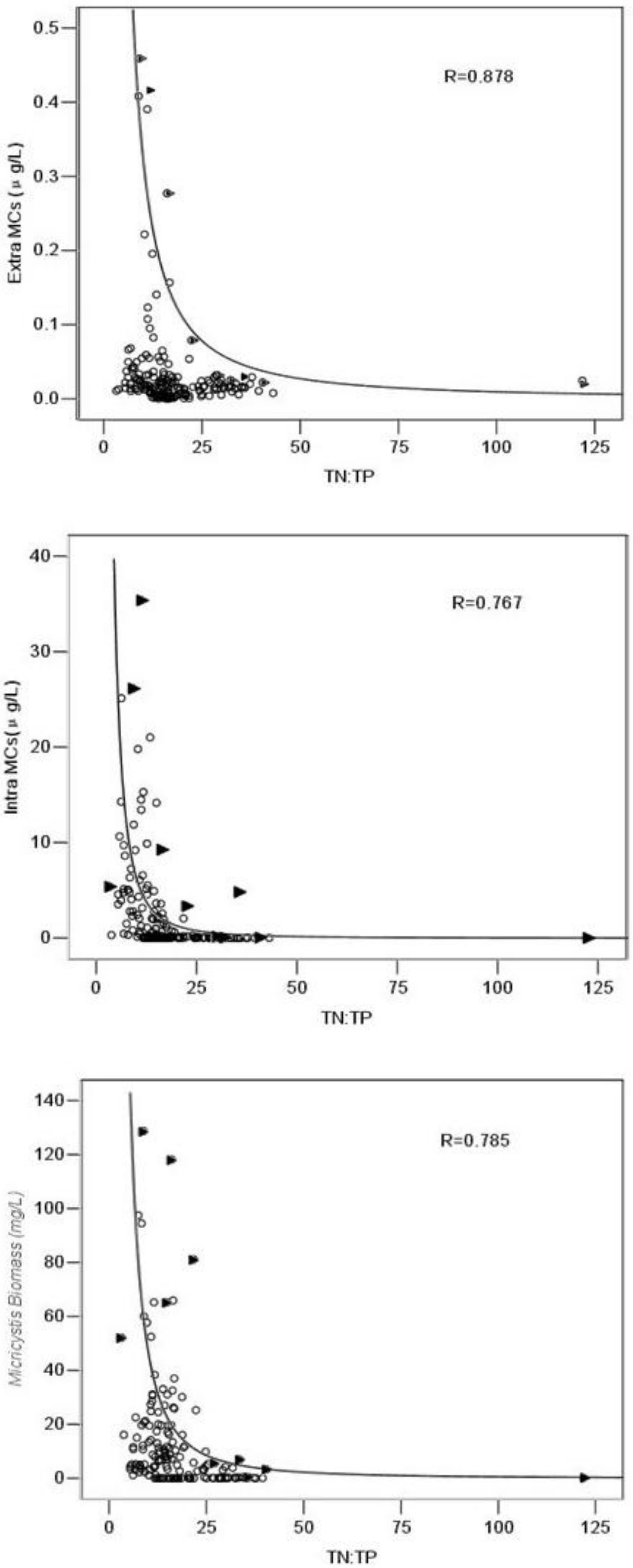

**FIGURE 10**. Relationships between TN:TP ratio and intra- or extracellular MC concentration or *Microcystis* biomass. Curves were estimated using IMR.

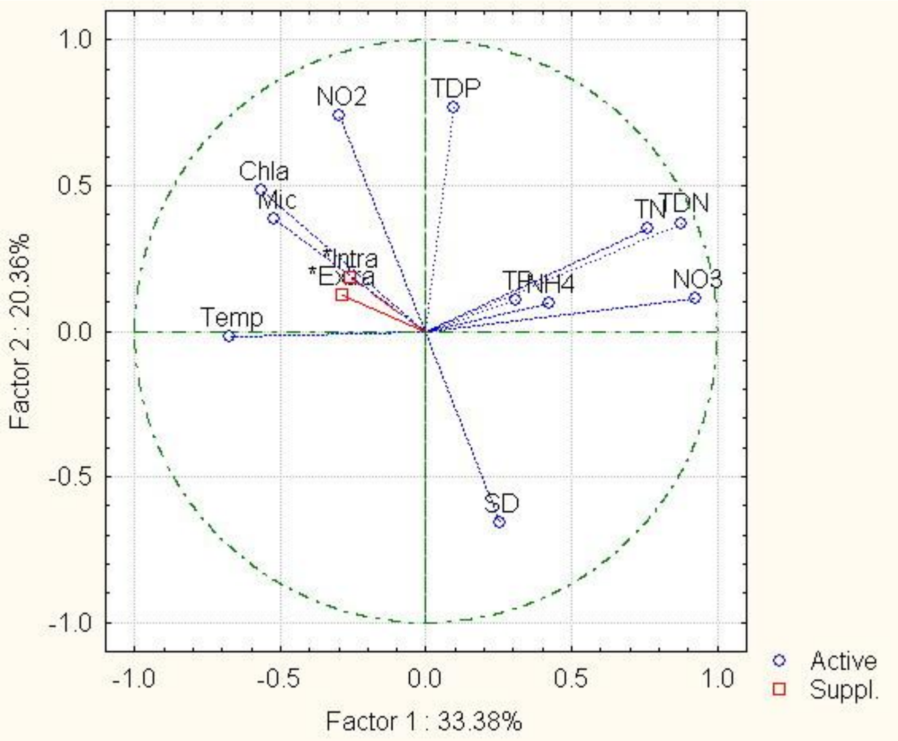

**FIGURE 11.** Scatter plot of first two components from a PCCA performed with 11 physiochemical or biological variation and MC variables from all samples (○, the active variables; □, the supplementary; Extra: extracellular MC content; Intra: intracellular MC content; Mic: *Microcystis* biomass; Temp: temperature; TN: total nitrogen; TP: total phosphorus; SD: Secchi depth).

More recently, a wider survey of eutrophic lakes showed that the TN:TP ratio was more useful to explain the variability of the MC concentration in water than was TP concentration[38]. In the present study, intra- and extracellular MCs had negative exponential relationships with TN:TP, and the maximum intra- and extracellular MCs values both occurred when TN:TP was <25, which was similar to results reported by Wu et al.[25] and Graham et al.[34]. The negative exponential decline in *Microcystis* biomass along the TN:TP gradient perhaps reflected superior competitive ability of *Microcystis* at a low ratio of TN:TP. Amano et al.[40] reported that a significant suppression of *Microcystis* growth of 70% could be achieved when the TN:TP ratios exceeded 21. Both TN and TP increased from August to October in our study, while the TN:TP ratio was relatively low in summer and autumn, probably because more P than N was released from the sediment during *Microcystis* blooms[25,41,42].

Multivariate analyses by principal component and classifying analysis indicate that intra- and extracellular MC concentrations have better correlations with biological factors (such as *Microcystis* biomass and chl-a ) than with physicochemical factors in our study. Among the physicochemical factors, temperature was an important variable closely related with MCs. MCs were negatively correlated with SD, suggesting that clear water may not favor MC production by cyanobacteria on one hand[34,43], and on the other hand, the accumulation of toxin-production cyanobacteria may lead to the decrease of SD.

During our study period, MC concentration was higher in summer and autumn than in other seasons, and both *Microcystis* biomass and MC concentrations reached peaks in October. Liu et al.[23] found that extracellular MC concentrations reached a peak in June (about 0.03 μg/L) and intracellular MCs concentrations reached a peak in July (about 3 μg/L) in a study period from November 2004 to October 2005 in Gonghu Bay. Thus, the peak concentrations of MCs were three to four times higher in 2008 than in 2005, indicating more serious cyanobacterial blooms and cyanotoxin contamination in Gonghu Bay recently.

In our study, the mean MC content in lake water was 11.8 μg/L in October and the maximum concentration reached as high as 35.8 μg/L, which is considerably higher than those of *Microcystis* blooms in other regions of the world[38,44,45]. The WHO has proposed a provisional guideline level of 1.0 μg/L for MC-LReq in drinking water and 20 μg/L in recreation water[46]. The MC-LReq value was used as a standard for assessing the risks associated with MCs. The intraperitoneal median lethal dose in mice for MC-RR and -YR was 5.0- and 2.5-fold higher, respectively, than that for MC-LR[47]; therefore, coefficients of 0.2 and 0.4 were used to convert MC-RR and -YR, respectively, into the MC-LR equivalent (MC-LReq). In the present study, the maximum MC concentration reached as high as 17 μg/L MC-LReq and 77.6% of the water samples was above the safety limit of 1 μg/L MC-LReq required for drinking water. As an important water source of Wuxi city, MC pollution in Gonghu Bay is a serious threat to people's health. Moreover, because MCs are chemically very stable and do not readily undergo proteolytic or hydrolytic attack[48], these toxins can finally end up in human food through the food chain[10,49], and the chronic impact of accumulated MCs on human health cannot be ignored[11].

## ACKNOWLEDGMENT

This research was supported by a fund from the Ministry of Science and Technology of the People's Republic of China (Grant No. 2007BAC26B02) and by a project from the Chinese Academy of Sciences (Grant No. KZCX1-YW-14-6-1). We would like to express our thanks to Dr. Meng Zhang for his kind assistance in the field. We also thank Dr. Dawen Zhang for very useful comments and suggestions on the manuscript.
